# Thermoregulatory Performance and Thermal Comfort Analysis of Phase-Change Fiber Seamless Knitted Fabrics

**DOI:** 10.3390/ma18235317

**Published:** 2025-11-25

**Authors:** Jingfeng Cheng, Lu Chang, Jiahui Fei, Zimin Jin, Mingtao Zhao

**Affiliations:** 1 College of Textile Science and Engineering (International Silk College), Zhejiang Sci-Tech University, Hangzhou 310000, China; cjf0823@163.com (J.C.); feijiahui1999@163.com (J.F.); 2 Shanghai Yijin Testing Technology Co., Ltd., Shanghai 200001, China; cherry@test-china.com; 3 Zhejiang Bangjie Holding Group Co., Ltd., Yiwu 322000, China; mtzhao@bangjie.cn

**Keywords:** phase-change fiber, thermal comfort, temperature-regulating fabrics

## Abstract

The escalating climate change and extreme weather conditions have highlighted the limitations of conventional textiles in body temperature regulation Phase-change thermoregulatory fibers can maintain constant temperature when environmental conditions fluctuate, effectively meeting human comfort requirements. To investigate the temperature-regulating properties of TempSolution viscose/cotton thermoregulatory yarn in seamless knitted fabrics, this study examined three different knitted fabric structures along with varying blend ratios and linear densities of TempSolution viscose/cotton yarn as experimental variables. According to a full factorial experimental design, eighteen fabric samples and corresponding garment prototypes were produced on a seamless circular knitting machine. Differential scanning calorimetry (DSC) and thermal manikin tests were conducted to compare the thermoregulatory performance and thermal comfort of seamless knits with different yarn compositions and structures. The results demonstrated that among the fabric structures, the 1 + 3 mock rib configuration exhibited superior thermal resistance and comfort properties. Regarding yarn types, the 50/50 TempSolution blend with 50 s yarn count showed relatively superior performance across all test parameters, providing a theoretical foundation for developing phase-change knitted fabric.

## 1. Introduction

Currently, with the intensification of global climate change and the increasing frequency of extreme weather events, thermal comfort management has become a critical challenge in the field of smart textiles. Conventional knitted garments rely on either thermal insulation or breathability for unidirectional temperature regulation. For instance, wool products achieve warmth primarily through their thermal insulation, Coolmaxenhances cooling performance by improving fabric breathability. However, these traditional approaches fail to dynamically respond to fluctuations in environmental temperature [1]. Phase change materials (PCMs) are a class of latent heat storage materials whose thermoregulation mechanism stems from their high phase-change enthalpy characteristics and dynamic thermal equilibrium regulation capabilities [2]. By forming and breaking molecular bonds, PCMs can efficiently store or release large amounts of energy [3,4]. When the ambient temperature rises, PCMs absorb and store substantial latent heat through phase transition, and when the temperature drops, they release heat to maintain a stable surface temperature. This property enables PCMs to act as an effective bridge between energy supply and demand, significantly improving energy efficiency [5]. Additionally, PCMs exhibit a broad range of phase transition temperatures. Microencapsulation [6] and nanoencapsulation of PCMs [7] have emerged as the preferred technique for PCM integration into textiles, ensuring durability and preventing PCM leakage. However, microencapsulated phase-change materials (MicroPCMs) have certain limitations. When the liquid phase-change material begins to crystallize at temperatures significantly below its designated melting point or within a broad temperature range where crystallization occurs, supercooling may take place, potentially leading to energy loss [8,9].

Phase-change materials (PCMs) have garnered significant attention in advanced textile applications, including medical textiles, military apparel, and intelligent textiles, owing to their exceptional dynamic thermal regulation capabilities. In the domain of medical textiles, Fu Wenliang et al. [10] engineered a chitosan-based composite temperature-responsive wound dressing incorporating keratinocyte growth factor-2 (KGF-2) mutants. This formulation undergoes a rapid liquid-to-solid transition at ambient temperatures exceeding 35 °C, exhibiting superior conformability, barrier properties, and moisture retention. Furthermore, it facilitates sustained drug release at the wound site, thereby enhancing tissue regeneration and accelerating wound healing. Regarding military applications, Gu et al. [11] developed microencapsulated PCMs using eicosane as the core material and a melamine–urea–formaldehyde (MUF) resin as the encapsulating shell. Subsequent polyaniline (PANI) deposition on the microcapsule surface yielded dynamic stealth materials (DSMs). The resultant textile demonstrated dual functionality, achieving a temperature reduction of 11.2 °C with a prolonged thermal regulation duration of 27 min, while simultaneously exhibiting a reduced infrared emissivity of 0.794. Infrared thermographic analysis confirmed the fabric’s effective infrared camouflage performance. In the context of intelligent textiles, Zhang et al. [12] proposed an innovative PCM-based cooling garment designed for miners operating in high-temperature underground environments. The garment employs a stratified PCM configuration, integrating 15 °C PCMs as the inner layer and 23 °C PCMs as the outer layer. Thermal manikin experiments and human trials revealed that the garment significantly enhanced heat dissipation, attenuated the rate of skin temperature increase, and improved thermal comfort perception under hot and humid conditions, thereby optimizing occupational safety and productivity.

Integrating PCMs with textiles offers transient warmth and cooling effects, effectively delaying discomfort [13]. Phase-change fibers (PCFs), also referred to as thermoregulated fibers or “air-conditioning fibers,” represent a novel class of functional fibers fabricated by integrating phase-change materials (PCMs) into a fibrous matrix [14,15]. These fibers exhibit temperature-regulating properties, mitigating discomfort caused by thermal fluctuations—such as perspiration or shivering—thereby providing enhanced wearing comfort across diverse climatic conditions. The heat absorption and release processes of PCFs are autonomous, reversible, and bidirectional [14]. When incorporated into knitted garments, PCFs facilitate autonomous microclimate regulation, effectively maintaining individual thermal comfort by dynamically adapting to body temperature variations.

Therefore, this study fabricates seamless knitted underwear by employing phase-change thermoregulatory fibers with varying blend ratios and linear densities in three distinct structural configurations. Through differential scanning calorimetry (DSC) and thermal manikin testing, we systematically investigate the influence of different fabric structures, yarn linear densities, and blend ratios on the thermoregulatory performance and thermal comfort of phase-change seamless knitted fabrics. This research provides valuable references and insights for the future development of thermoregulatory knitted apparel products.

## 2. Materials and Methods

### 2.1. Materials

#### 2.1.1. Yarn Selection

The face yarns for this study were selected from Qingdao Nehemiah Biotechnology Co., Ltd. (Qingdao, China), including 18.45 tex (32 s) and 11.81 tex (50 s) yarns with 30% thermoregulatory yarn + 70% pure cotton yarn and 50% thermoregulatory yarn + 50% pure cotton yarn. Conventional pure cotton yarn was added as a blank control group to compare the differences between thermoregulatory fabrics and regular pure cotton fabrics. The yarn fabric specification table is presented in Table 1. The PCM is a bio-based phase-change material, with raw materials 100% derived from natural oils, primarily palm oil, palm kernel oil, and coconut oil. The cotton yarn was spun using the ring spinning process, with a twist level of 90 twists per 10 cm.

#### 2.1.2. Fabric Structure Design

Different fabric structures have significant impacts on the thermoregulatory performance, thermal comfort properties, and wearability of temperature-regulating knitted garments. Based on the varying thermal comfort requirements and physiological characteristics of different body parts, three typical fabric structures were designed and selected: plain knit structure, 1 + 1 rib structure, and 1 + 3 mock rib structure.

#### 2.1.3. Establishment of the Sample Scheme

This research examines the influence of fabric manufacturing techniques on the temperature regulation performance and thermal comfort characteristics of phase-change thermoregulatory fabrics, based on six face yarn types and three fabric structure types. The experimental group consists of six levels of face yarn types and three levels of fabric structures. Following a full factorial experimental design, eighteen fabric samples were produced. All fabric specimens were knitted using a Santoni SM8-TOP2 MP seamless circular knitting machine (Santoni (Shanghai) Knitting Machinery Co., Ltd., Shanghai, China), maintaining identical knitting parameters throughout the manufacturing process: a 1:1 feed ratio of face yarn to backing yarn, 28-gauge needles per inch, 13-inch cylinder diameter, 1152 needles, and consistent stitch cam height positioning. The sample specifications are presented in Table 2.

### 2.2. Methods

#### 2.2.1. DSC Testing and Analysis

The DSC represents a thermal analysis instrument that achieves analysis of thermal response characteristics of samples under programmed temperature control through measurement of heat flow differences between the sample and reference material. DSC testing was conducted in accordance with ASTM D3418 standard [16] method, with the temperature range adjusted to accommodate the characteristics of the phase-change materials.

Experimental apparatus: Q2000 differential scanning calorimeter (Zhejiang JinHong Instrument Equipment Co., Ltd., Hangzhou, China).

Experimental procedure: Approximately 10 mg samples were taken from each of the aforementioned 18 fabric specimens, then cut into small pieces to completely fit into Mettler aluminum crucibles. The samples were placed in the DSC instrument with an ensured appropriate and uniform quantity. Testing conditions were set as follows: starting from −40 °C, heating to 100 °C at a rate of 10 °C/min, then cooling back to −40 °C at the same rate. During testing, the instrument recorded the heat flow changes in samples, generating DSC curves.

#### 2.2.2. Thermal Resistance and Thermal Comfort Performance Testing Based on a Thermal Manikin

Conventional thermal resistance testing methods focus solely on localized fabric measurements, exhibiting certain limitations. This study employs a thermal manikin to simulate realistic human body conditions, enabling comprehensive evaluation of the heat and moisture transfer processes occurring at the body–clothing–environment interface. For the 18 phase-change thermoregulatory knitted garment prototypes, we systematically measured both the clothing thermal resistance and predicted mean vote (PMV) of whole-body thermal sensation. The investigation provides an in-depth analysis of how face yarn composition and fabric structure influence the garments’ thermal comfort performance, thereby establishing scientific foundations for the design and optimization of phase-change thermoregulatory knitted garments.

1. Experimental Garments

To standardize the static air layer volume between the garment and human body while minimizing the influence of garment tension variations on thermal manikin testing, all experimental garments were manufactured under strict dimensional control. The garments were produced in men’s size 170/80A (Chinese standard sizing system [17], with the phase-change thermoregulatory fabrics listed in Table 1 being fabricated into complete garments designated sequentially as B01 through B18. Moreover, three independent fabric samples (n = 3) were produced for each garment prototype B01-B18, resulting in a total of 54 samples. The selection of this sample size (n = 3) aligns with the common practice for performance testing in the field of intelligent thermoregulatory textiles.

2. Experimental Equipment

(1) Thermal Manikin System

**Figure 1:**
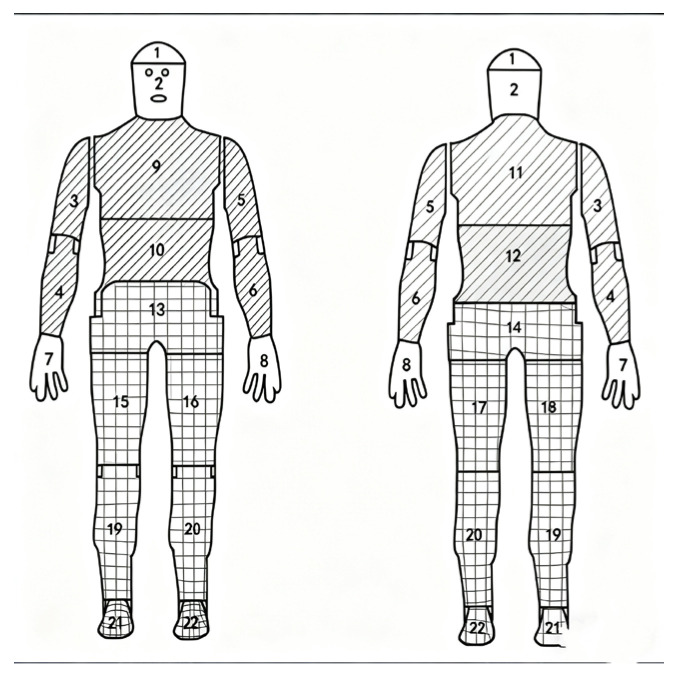
Zoning diagram of thermal manikin. The diagonally hatched region represents the upper garment, while the cross-hatched (grid) region represents the lower garment.

The thermal manikin (Hillerød, Denmark) used in the experiment was manufactured by PT TEKNIK in Denmark, model Robert. The manikin has a height of 172 cm with a surface area of approximately 1.68 m^2^. The body is divided into 22 zones, including head, face, left upper arm front/back, etc. The zoning diagram is shown in Figure 1. In this study, zones 1–12 (including head, chest, abdomen, back, and arms) were classified as Group A for upper garment evaluation; zones 13–22 (including waist, legs, and feet) were designated as Group B for lower garment assessment; while all 22 zones combined constituted the full ensemble evaluation.

(2) Artificial Climate Chamber

The artificial climate chamber can achieve stable and adjustable temperature and humidity in the experimental space to simulate natural climate conditions. The chamber’s temperature control range is −10 to 40 °C with an adjustment accuracy of ±1 °C, humidity adjustment range is 45% to 75% with an adjustment accuracy of ±10% RH, features top air supply and bottom air return, with wind speed controlled at ≤0.1 m/s. The thermal manikin was placed in the center of the artificial climate chamber. Testing commenced after the temperature and humidity parameters reached and stabilized at their preset values, followed by a one-hour equilibrium period to ensuring uniformity and stability of the environmental conditions.

##### Clothing Thermal Resistance Testing and Analysis

1. Experimental Procedure

(1) Before testing, 18 types (comprising 54 sets) of knitted garments were placed under standard atmospheric conditions (temperature: 20 ± 2 °C, relative humidity: 65 ± 4%) for 12 h of conditioning. The operation panel of the artificial climate chamber was opened, and parameters were set according to ISO 15831-2004 “Measurement of Thermal Insulation by Means of a Thermal Manikin” [18]: temperature was set to 22 °C with a fluctuation range of ±1 °C, and relative humidity was set to 50% with a fluctuation range of ±10%.

(2) After the environmental temperature and humidity parameters stabilized, the thermal manikin was suspended on the mounting frame in a standing posture, elevated 5 cm above the ground to eliminate heat conduction losses.

(3) Activate the thermal manikin, first test the “Nude” baseline thermal resistance value. The steps are as follows: select PI (constant temperature mode), set the surface temperature of all body segments to 34 °C with a control accuracy of ±0.1 °C, initiate the heating program, wait for the test readings to stabilize, and click “Analyze” to automatically record the values.

(4) Dress the thermal manikin with the sample garment, repeat step (3), test the “Clothing” thermal resistance value, click “Analyze”, import the nude thermal resistance value, and automatically record the data.

2. Thermal Resistance Calculation Method

Clothing thermal resistance is divided into: total clothing thermal resistance (*I_t_*), effective clothing insulation (*I_clu_*), and air layer resistance (*I_a_*). The total thermal resistance (*I_t,i_*) for the i-th segment of the manikin body can be calculated using Formula (1).

(1) $$I_{t,i}=\frac{T_{sk,i}i-T_{a}}{0.155\times H_{i}}$$

Among them, *T_sk_*_,*i*_ represents the skin temperature of the i-th segment of the dummy’s body, in °C; *T_a_* represents the ambient temperature, in °C. *H_i_* is the heating flow rate of the i-th section of the dummy body, W/m ^2^. The constant 0.155 m^2^·°C/W/clo is the conversion factor between the international unit of thermal resistance and the clo value (1 clo = 0.155 m^2^·°C/W).

The total body thermal resistance value was calculated using the parallel method according to Equation (2).

(2) $$I_{t}=\frac{\left((\sum f_{i}T_{sk,i})-T_{a}\right)}{0.155\times \sum f_{i}H_{i}}$$

Among them, *f_i_* is the ratio of the surface area of the i-th section of the dummy to the total surface area.

The effective clothing insulation represents the additional thermal insulation compared to the nude condition and can be calculated using Equation (3).

(3) $$I_{clu}=I_{t}-I_{a}$$

Among them, *I_a_* represents the thermal resistance of the air layer surrounding the skin surface when exposed. The result of this experiment is the relative thermal resistance value of the clothing.

##### PMV Testing and Analysis

1. Experimental Procedure

(1) Prior to testing, the 18 knitted garments were placed under standard atmospheric conditions for temperature and humidity conditioning for 12 h. The operation panel of the artificial climate chamber was opened, and parameters were set according to ISO 7730: 2005 [19]: temperature was set to 22 °C with a fluctuation range of ±1 °C, and relative humidity was set to 50% with a fluctuation range of ±10%.

(2) After the environmental temperature and humidity parameters stabilized, the thermal manikin was suspended on the mounting frame in the identical posture as adopted during the thermal resistance testing, thereby preventing potential influences on clothing thermal comfort induced by postural variations.

(3) Select the comfort mode, set the surface temperature of all body segments to 34 °C with a control accuracy of ±0.1 °C, import the previously saved clothing thermal resistance values for each group, wait for the thermal manikin to reach thermal equilibrium, then calculate the PMV test value (hereinafter referred to as PMV value).

##### PMV Experimental Calculation Method

Based on previous research findings, Professor Fanger proposed the PMV index for thermal comfort prediction through extensive human physiological experiments combined with the human heat balance equation. This index provides a scientific basis for thermal comfort evaluation by quantifying comfort levels in thermal environments. Through quantitative analysis of numerical simulation data, it further defines comfort level classifications for different PMV ranges, as shown in Table 3, serving as a critical reference for thermal comfort testing.

PMV can be calculated by Equations (4)–(8).

(4) PMV=[0.303·exp(−0.306·M)+0.028

(5) $$\left\{\left(M-W)-3.05\cdot 10^{-3}[5.733\;-\;6.99(M\;-\;W)\;-\;p_{a}]\;-\;0.42[(M\;-\;W)\;-\;58.15]\;-1.7\cdot 10^{-5}\cdot M(5867-p_{a})-0.0014\cdot M(34-t_{a})\;\;\;\;\;\;-3.96\cdot 10^{-8}\cdot f_{\mathrm{cl}}[{(t_{\mathrm{cl}}+273)}^{4}-{({\bar{t}}_{r}+273)}^{4}]-f_{\mathrm{cl}}\cdot h_{c}(t_{\mathrm{cl}}-t_{a}\right)\right\}$$

(6) $$t_{\mathrm{cl}}=35.7\;-\;0.028(M\;-\;W)-I_{\mathrm{cl}}\left\{3.96\cdot 10^{-8}\cdot f_{\mathrm{cl}}[{(t_{\mathrm{cl}}+273)}^{4}-{(t_{r}+273)}^{4}]-f_{\mathrm{cl}}\cdot h_{c}(t_{\mathrm{cl}}\;-\;t_{a})\right\}$$

(7) $$h_{c}=\left\{\begin{array}{c} 2.38\cdot {\left|t_{\mathrm{cl}}\;-\;t_{a}\right|}^{0.25}\;\;\;\;\;\;for\;\;\;\;\;2.38\cdot {\left|t_{\mathrm{cl}}\;-\;t_{a}\right|}^{0.25}<12.1\cdot \sqrt{v_{\mathrm{ar}}}\\ 12.1\cdot \sqrt{v_{\mathrm{ar}}}\;\;\;\;\;\;\;\;\;\;\;\;\;for\;\;\;\;\;2.38\cdot {\left|t_{\mathrm{cl}}\;-\;t_{a}\right|}^{0.25}<12.1\cdot \sqrt{v_{\mathrm{ar}}} \end{array}\right.\;\;\;$$

(8) $$f_{cl}=\left\{\begin{array}{c} 1.00+1.290\;I_{cl}\;\;for\;\;I_{cl}\leq 0.078\;m^{2}\cdot K/W\\ 1.05+0.645\;I_{cl}\;\;for\;\;I_{cl}>0.078\;m^{2}\cdot K/W \end{array}\right.\;\;\;$$

*M*: Metabolic heat production per unit body surface area (W/m^2^). *W*: Effective mechanical power (W/m^2^). *I_cl_*: Thermal resistance of clothing (m^2^·K/W). *f_cl_*: Clothing area factor (dimensionless). *t_a_*: Air temperature (°C). ${\bar{t}}_{r}$: Mean radiant temperature (°C). *v_ar_*: Relative air velocity (m/s). *p_a_*: Water vapor partial pressure (Pa). *h_c_*: Convective heat transfer coefficient (W/(m^2^·K)). *t_cl_*: Clothing surface temperature (°C). The specific parameters were as follows: metabolic rate (M) = 1.2 met, ambient temperature (ta) = 22 °C, and relative air velocity (var) = 0.10 m/s.

##### Data Analysis Methods

To quantitatively assess the significant effects of fabric structure and blend ratio on fabric thermal resistance and thermal comfort (PMV), this study conducted a comprehensive statistical analysis based on all 54 independent samples’ raw observations using IBM SPSS Statistics 27 software. After verifying that the data met the assumptions of normality (Shapiro–Wilk test, *p* > 0.05) and homogeneity of variances (Levene’s test, *p* > 0.05) (see Supplementary Table S8), a full factorial two-way ANOVA model was employed to examine the main effects of the two fixed factors—fabric structure and blend ratio—as well as their interaction effects. The significance threshold was set at α = 0.05. For main effects that reached statistical significance, post hoc multiple comparisons were performed using Duncan’s test at *p* = 0.05, which controls Type I error through its stepwise procedure without requiring additional correction.

## 3. Results and Analysis

### 3.1. DSC Experimental Results and Analysis

**Figure 2:**
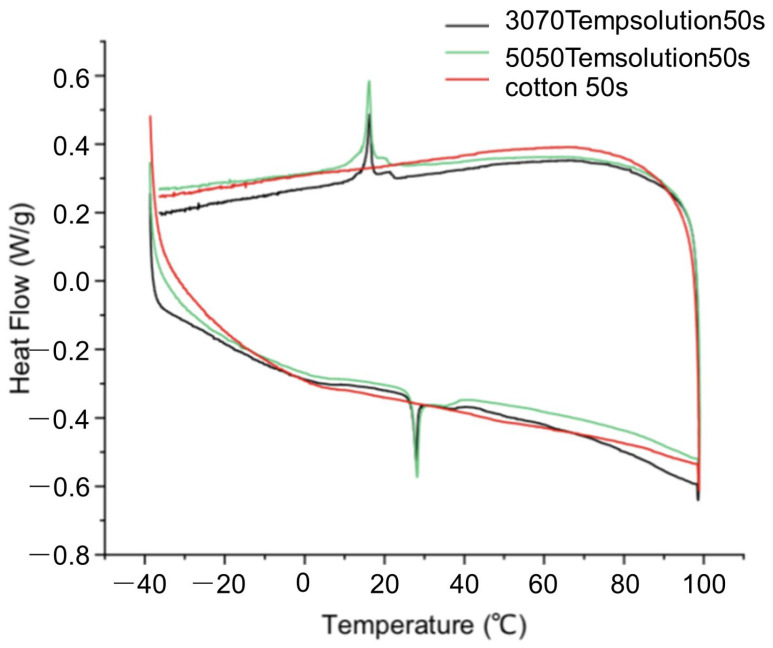
DSC thermograms.

The test results for the 50 s yarn are presented in Figure 2, and the graphical analysis data are presented in Table 4.

Analysis was conducted on the DSC differential scanning calorimetry test result curves shown in Figure 2. Since conventional pure cotton yarn did not exhibit characteristic endothermic/exothermic peaks, it was excluded from the analysis. The analysis results are presented in Table 4. When ambient temperature changes, the heating phase transition range of 3070 TempSolution thermoregulatory yarn 50 s is 21.77–31.66 °C, and the cooling phase transition range is 23.37–9.02 °C; the heating phase transition range of 5050 TempSolution thermoregulatory yarn 50 s is 20.18–39.73 °C and the cooling phase transition range is 23.58–3.71 °C. Further statistics reveal that all thermoregulatory yarns exhibit higher enthalpy change values during the heating process compared to the cooling process, indicating higher energy storage/release efficiency. The above results demonstrate that both types of thermoregulatory yarns possess excellent temperature regulation capabilities, and the phase transition triggering temperature ranges align with the human physiological comfort zone. However, a certain degree of energy loss is observed in the yarns, primarily attributed to the common “supercooling” phenomenon in phase-change materials. Owing to the presence of triglycerides in the composition of natural oil-based phase-change materials, their varying chain length molecules mutually interfere during crystallization, creating steric hindrance that impedes the formation of an ordered crystal lattice. This results in a generally greater degree of supercooling compared to petroleum-based materials, which has been experimentally verified in natural fatty acid systems such as palmitic acid [20].

### 3.2. Thermal Resistance Experimental Results and Analysis

According to the testing standards, 18 sets of phase-change thermoregulatory knitted garments were tested, with thermal resistance values recorded separately for upper garments, lower garments, and full ensembles. Specific results are shown in Table 5. SPSS ANOVA yielded tests of between-subjects effects for the garment ensemble, upper garment, and lower garment. The tests of between-subjects effects indicated that Factor A (face yarn type) had a highly significant influence on the thermal resistance of the garment ensemble (F(5,36) = 28.874, *p* < 0.001, η^2^p = 0.800), upper garment (F(5,36) = 23.768, *p* < 0.001, η^2^p = 0.768), and lower garment (F(5,36) = 20.668, *p* < 0.001, η^2^p = 0.742; see Supplementary Table S1). Similarly, Factor B (fabric structure) also demonstrated a highly significant effect on thermal resistance across the ensemble (F(2,36) = 31.059, *p* < 0.001, η^2^p = 0.633), upper garment (F(2,36) = 35.041, *p* < 0.001, η^2^p = 0.661), and lower garment (F(2,36) = 34.116, *p* < 0.001, η^2^p = 0.655; see Supplementary Table S1). However, the interaction between yarn type and fabric structure (A × B) showed no significant effect on the thermal resistance of the ensemble (F(10,36) = 1.121, *p* = 0.374, η^2^p = 0.237), upper garment (F(10,36) = 0.883, *p* = 0.557, η^2^p = 0.197), or lower garment (F(10,36) = 1.015, *p* = 0.450, η^2^p = 0.220). These results suggest that the contributions of yarn type and fabric structure to thermal resistance are relatively independent. Duncan’s test further revealed that for the thermal resistance of upper garments, lower garments, and ensembles, the ‘50/50 TempSolution 50 s’ yarn and the 1 + 3 mock rib structure consistently outperformed all other yarn and structure types, forming the top performance tier (see Duncan’s test in Supplementary Table S3).

**Figure 3:**
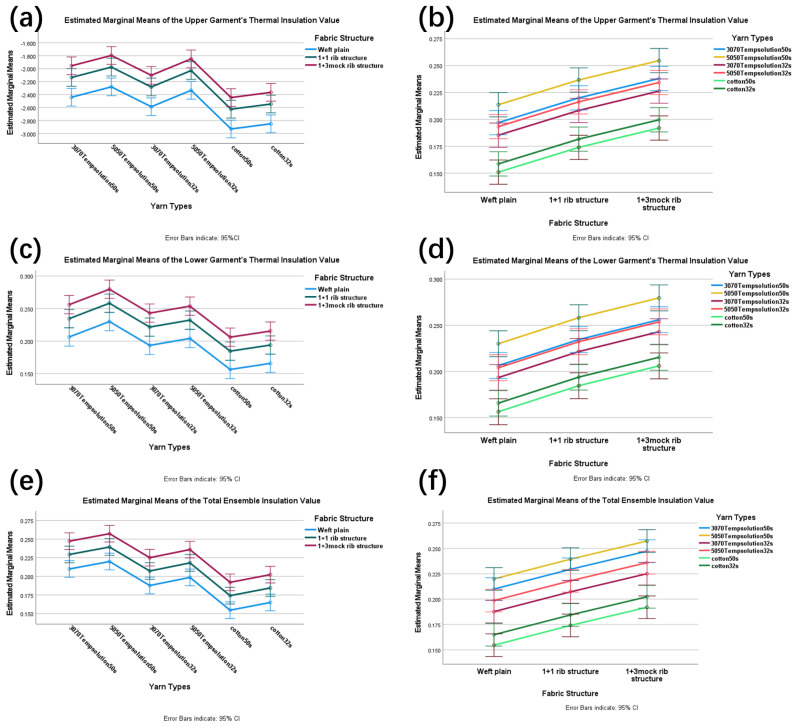
Estimated marginal mean profile plots of thermal resistance values for face fabric type and organizational structure of upper garment (**a**,**b**), lower garment (**c**,**d**), and full ensemble (**e**,**f**). Error bars: 95% confidence interval.

Figure 3 presents the estimated marginal mean profiles of thermal resistance for face yarn types and fabric structures across the garment ensemble, upper garment, and lower garment. When face yarn type was considered as the independent variable, significant differences were observed in all three garment categories, with the 1 + 3 mock rib structure demonstrating superior thermal resistance compared to other structures. Similarly, when fabric structure served as the independent variable, distinct variations were found across all garment types, where the 50/50 TempSolution 50 s yarn outperformed all other yarn types. These findings are consistent with the results obtained from Duncan’s test.

### 3.3. PMV Test Results and Analysis

Table 6 shows the PMV test results of phase-change thermoregulatory fiber garments. SPSS ANOVA yielded tests of between-subjects effects for the garment ensemble, upper garment, and lower garment. For Factor A (face yarn type), its effect on fabric PMV reached a highly significant level across all garment types: ensemble (F(5,36) = 39.112, *p* < 0.001, η^2^p = 0.845), upper garment (F(5,36) = 28.167, *p* < 0.001, η^2^p = 0.796), and lower garment (F(5,36) = 20.290, *p* < 0.001, η^2^p = 0.738; see Supplementary Table S2). Similarly, Factor B (fabric structure) also demonstrated a highly significant influence on PMV values for the ensemble (F(2,36) = 64.079, *p* < 0.001, η^2^p = 0.781), upper garment (F(2,36) = 39.073, *p* < 0.001, η^2^p = 0.685), and lower garment (F(2,36) = 33.388, *p* < 0.001, η^2^p = 0.650; see Supplementary Table S2).

Unlike the findings for thermal resistance, the interaction between yarn type and fabric structure (A × B) exhibited body region specificity for PMV. This interaction showed no significant effect on PMV values of the lower garment (F(10,36) = 0.802, *p* = 0.628, η^2^p = 0.182). However, it demonstrated significant interactive effects on both the ensemble (F(10,36) = 2.562, *p* = 0.019, η^2^p = 0.416) and upper garment (F(10,36) = 2.391, *p* = 0.027, η^2^p = 0.399). To further investigate these significant interactions, simple effects analyses were conducted for the garment ensemble and the upper garment.

Simple effects analysis of the upper garment revealed that the combination of “50/50 temperature-regulating yarn 50 s with 1 + 3 mock rib structure” achieved relatively optimal thermal comfort, with its PMV value of −1.940 (95% CI: −2.194, −1.686) being closest to the neutral comfort zone. Furthermore, this yarn performed significantly better in this structure than in plain weave (PMV = −2.840, MD = 0.900, *p* < 0.001) and 1 + 1 rib (PMV = −2.360, MD = 0.420, *p* = 0.023). Additionally, within rib structures, all temperature-regulating yarns outperformed cotton yarns (e.g., 50/50 temperature-regulating yarn 50 s vs. cotton 50 s, MD = 1.340, *p* < 0.001). These findings are consistent with the results presented in the estimated marginal mean plots for upper garments (c–d), collectively demonstrating a significant synergistic effect between yarn type and fabric structure (Among them, CL is the confidence interval, and MD is the mean difference.

**Figure 4:**
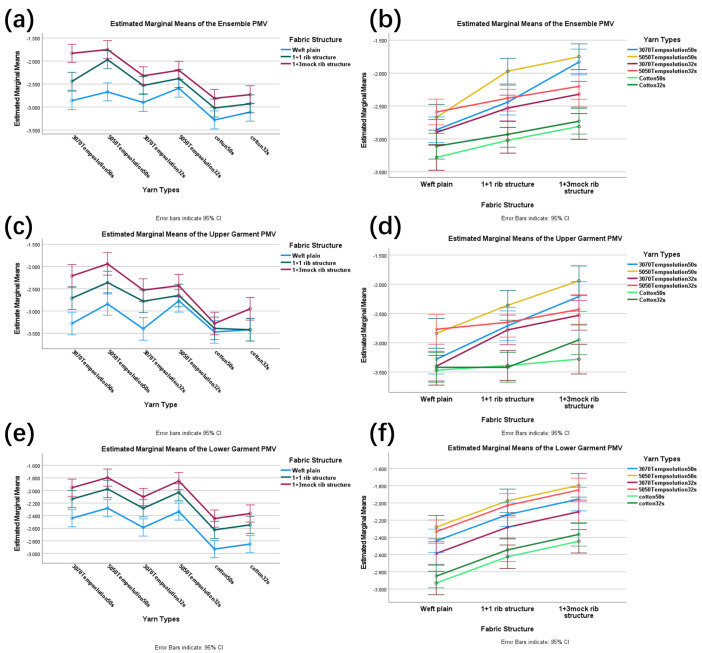
Estimated marginal mean profile plots of PMV values for face fabric type and organizational structure of full ensemble (**a**,**b**), upper garment (**c**,**d**), and lower garment (**e**,**f**). Error bars: 95% confidence interval.

Simple effects analysis of the garment ensemble demonstrated that the combination of “50/50 temperature-regulating yarn 50 s with 1 + 3 mock rib structure” achieved relatively optimal thermal comfort, with a PMV value of −1.750 (95% CI: −1.946, −1.554) being closest to the neutral zone. This yarn performed significantly better in this structure than in plain weave (PMV = −2.670, MD = 0.920, *p* < 0.001) and 1 + 1 rib (PMV = −1.970, MD = 0.220, *p* = 0.023). Moreover, within rib structures, all temperature-regulating yarns substantially outperformed cotton yarns (e.g., 50/50 temperature-regulating yarn 50 s vs. cotton 50 s, MD = 1.060, *p* < 0.001). These results are consistent with those presented in Figure 4 the estimated marginal mean plots for the garment ensemble (a-b), collectively providing evidence for a significant synergistic effect between yarn type and fabric structure.

In contrast to the upper garment and ensemble, the interaction between face yarn type and fabric structure did not significantly affect the PMV values of the lower garment. Figure 4e,f indicate that among the various face yarn types, the 50/50 temperature-regulating yarn 50 s demonstrated relatively optimal performance with a PMV value of −2.02 (95% CI: −2.14, −1.90). Similarly, among the fabric structures, the 1 + 3 mock rib exhibited the best performance with a PMV value of −2.09 (95% CI: −2.17, −1.99). These findings align with the trends observed for the upper garment and ensemble.

Duncan’s post hoc test results clearly demonstrated that across all samples, the ‘50/50 TempSolution 50 s’ yarn and ‘1 + 3 mock rib structure formed the top performance tier in terms of both thermal resistance and thermal comfort properties, representing the relatively optimal combination. Complete data are provided in Supplementary Tables S3–S6.

### 3.4. Discussion and Analysis

#### 3.4.1. Interpretation of the Mechanisms Underlying the Findings

The experimental results clearly demonstrated that among all samples, the fabric knitted with the 1 + 3 mock rib structure using 50/50 TempSolution yarn (50 s) exhibited relatively superior performance in both thermal resistance and thermal comfort (PMV). This finding is highly consistent with the conclusions of Zhou et al. [21], whose research confirmed that rib-based structures, due to their unique three-dimensional loop arrangement, can form a thicker and more stable air layer, thereby significantly improving the fabric’s thermal resistance. Furthermore, the 1 + 3 mock rib structure demonstrated the maximum measured thickness and fabric weight per unit area (see Supplementary Table S7: Fabric Thickness/Weight Table). Its bulky configuration facilitates the formation of thicker static air layers and enhances air entrapment. This mechanism aligns with findings from Raja [22], collectively illustrating how the enhanced air layer achieved through the bulky structure of the 1 + 3 mock rib improves thermal insulation performance.

In terms of material characteristics, the 50 s yarn contributes to a denser and more uniform fabric structure compared to the 32 s temperature-regulating yarn. This structural advantage may promote the formation of stable static air layers and improve the encapsulation efficiency of phase-change materials. The present study provides valuable theoretical and data-driven support for future development of intelligent temperature-regulating garments based on phase-change materials.

This study also revealed that the interaction between fabric structure and face yarn type had no significant effect on the thermal resistance of the samples. However, for PMV, this interaction significantly influenced both the upper garments and full ensembles, but not the lower garments. This discrepancy can be attributed to the fundamental nature of these parameters: thermal resistance is a straightforward physical property, whereas PMV represents a complex, integrated physiological perception influenced by multiple factors.

The significant interactive effects on PMV for upper garments and ensembles, but not for lower garments, align with Naeem, J.’s [23] observation that clothing exhibits distinct thermal-moisture properties across different body regions. The torso, being more sensitive than the lower limbs and serving as the body’s core heat-producing area, possesses a more complex microclimate, resulting in pronounced interactive effects. These findings provide a theoretical basis for differentiated zoning strategies in intelligent garment design.

#### 3.4.2. Limitations and Future Prospects

(1) DSC testing was conducted only on 50 s yarn; future studies should include comprehensive characterization of all yarn counts. The DSC results revealed significant supercooling in the phase-change material, indicating inherent initial energy loss in practical thermal energy cycling efficiency. Since only one thermal cycle was performed without laundering tests, the long-term cycling stability of the material requires further verification.
(2) The experiments were conducted solely under standard 22 °C conditions without testing in colder environments. The practical effectiveness in colder climates may be diminished, necessitating future performance evaluations under broader temperature ranges.
(3) For thermal resistance (ensemble/upper garment/lower garment) and PMV (lower garment), the interaction effects showed large effect sizes (η^2^p > 0.14) but failed to reach statistical significance (*p* > 0.05). This discrepancy may stem from the limited sample size (n = 3), which, although conforming to conventional textile testing standards, might have constrained the detection of subtle differences. Future studies should increase the sample size to enhance statistical power.

## 4. Conclusions

This study conducted DSC tests on two types of TempSolution phase-change thermoregulatory fibers with different blend ratios. Thermal manikin testing and analysis were performed on six face yarn types to investigate the effects of yarn type and fabric structure on the functional performance of 18 kinds of phase-change thermoregulatory seamless knitted fabrics. The study focused on thermal resistance and PMV values, yielding the following conclusions. DSC differential scanning calorimetry tests revealed that TempSolution phase-change thermoregulatory fibers exhibited significant heat absorption/release phenomena during both heating and cooling processes. The temperature range of the phase transition coincided with the human physiological comfort zone. In the thermal resistance tests, both the face yarn type and the organizational structure exerted highly significant effects on thermal resistance values. Among the face yarn types, the 5050 TempSolution thermoregulatory yarn 50 s demonstrated the highest thermal resistance, significantly outperforming the other five yarn types. Regarding organizational structures, the 1 + 3 mock rib structure exhibited the highest thermal resistance, followed by the 1 + 1 rib structure, while the plain knit structure showed the lowest values. In the PMV testing, both face yarn type and organizational structure significantly influenced thermal comfort performance. Among all samples tested in this study, the 50/50 TempSolution temperature-regulating yarn (50 s) demonstrated the relatively optimal PMV value, being the closest to the comfort zone. Among organizational structures, fabrics knitted with the 1 + 3 mock rib structure exhibited the highest PMV values, followed by the 1 + 1 rib structure, with the plain knit structure yielding the lowest values—consistent with the thermal resistance findings. In summary, the 1+3 mock rib structure demonstrated exceptional performance in both thermal resistance and thermal comfort properties. Among the face yarn types, the 5050 TempSolution thermoregulatory yarn 50 s consistently achieved optimal results across all evaluation metrics. The findings of this study offer a scientific foundation for the functional design and performance optimization of phase-change temperature-regulating knitted clothing.

## Figures and Tables

**Table 1:** Yarn fabric specification table.

Yarn Type	Yarn Blend Ratio	Yarn Count
3070 TempSolution yarn 50 s	30% PCM Viscose + 70% Cotton	11.81 tex (50 s)
3070 TempSolution yarn 32 s	30% PCM Viscose + 70% Cotton	18.45 tex (32 s)
5050 TempSolution yarn 50 s	50% PCM Viscose + 50% Cotton	11.81 tex (50 s)
5050 TempSolution yarn 32 s	50% PCM Viscose + 50% Cotton	18.45 tex (32 s)
Cotton Yarn 50 s	100%Cotton	11.81 tex (50 s)
Cotton Yarn 32 s	100% Cotton	18.45 tex (32 s)

**Table 2:** The combination and sample number of six face yarn types and three fabric structure types.

Sample Number	A (Yarn Type)	B (Fabric Structure)
1#	3070TempSolution yarn 50 s	Weft plain
2#	3070 TempSolution yarn 50 s	1 + 1 rib structure
3#	3070 TempSolution yarn 50 s	1 + 3 mock rib structure
4#	5050 TempSolution yarn 50 s	Weft plain
5#	5050 TempSolution yarn 50 s	1 + 1 rib structure
6#	5050 TempSolution yarn 50 s	1 + 3 mock rib structure.
7#	3070 TempSolution yarn 32 s	Weft plain
8#	3070 TempSolution yarn 32 s	1 + 1 rib structure
9#	3070 TempSolution yarn 32 s	1 + 3 mock rib structure
10#	5050 TempSolution yarn 32 s	Weft plain
11#	5050 TempSolution yarn 32 s	1 + 1 rib structure
12#	5050 TempSolution yarn 32 s	1 + 3 mock rib structure
13#	Cotton Yarn 50 s	Weft plain
14#	Cotton Yarn 50 s	1 + 1 rib structure
15#	Cotton Yarn 50 s	1 + 3 mock rib structure
16#	Cotton Yarn 32 s	Weft plain
17#	Cotton Yarn 32 s	1 + 1 rib structure
18#	Cotton Yarn 32 s	1 + 3 mock rib structure

**Table 3:** Relationship between PMV values and human thermal sensation [17].

PMV Value	−	−2	−1	0	1	2	3
Human Thermal Sensation	Very Cold	cold	Slightly Cool	Neutral	Slightly Warm	Warm	hot

**Table 4:** Analysis of DSC results of the 50 s yarn samples.

Yarn Raw Materials	Phase Transition Process	Phase Transition Temperature Range/°C	Peak Temperature/°C	Enthalpy Value/(J/g)
3070TempSolution 50 s	Heating Process	21.77~31.66	27.89	2.973
3070TempSolution 50 s	Cooling Process	23.37~9.02	16.27	1.677
5050TempSolution 50 s	Heating Process	20.18~39.73	28.15	4.132
	Cooling Process	23.58~3.71	16.17	3.828

**Table 5:** Thermal resistance test results of phase-change thermoregulatory fiber garments.

Sample Garment Number	Upper Garment/(m^2^·K/W)	Lower Garment/(m^2^·K/W)	Set/(m^2^·K/W)
B01	0.191 ± 0.010	0.198 ± 0.009	0.193 ± 0.028
B02	0.215 ± 0.013	0.227 ± 0.010	0.217 ± 0.019
B03	0.249 ± 0.012	0.272 ± 0.010	0.259 ± 0.011
B04	0.203 ± 0.018	0.214 ± 0.011	0.206 ± 0.018
B05	0.240 ± 0.012	0.266 ± 0.015	0.241 ± 0.005
B06	0.262 ± 0.048	0.288 ± 0.011	0.267 ± 0.015
B07	0.190 ± 0.006	0.199 ± 0.013	0.192 ± 0.009
B08	0.207 ± 0.010	0.220 ± 0.021	0.209 ± 0.009
B09	0.223 ± 0.008	0.239 ± 0.012	0.226 ± 0.018
B10	0.198 ± 0.008	0.210 ± 0.017	0.200 ± 0.014
B11	0.222 ± 0.016	0.235 ± 0.011	0.223 ± 0.014
B12	0.224 ± 0.022	0.245 ± 0.017	0.230 ± 0.019
B13	0.151 ± 0.009	0.163 ± 0.012	0.155 ± 0.007
B14	0.174 ± 0.011	0.186 ± 0.026	0.175 ± 0.013
B15	0.192 ± 0.010	0.198 ± 0.013	0.192 ± 0.004
B16	0.166 ± 0.016	0.172 ± 0.010	0.167 ± 0.008
B17	0.179 ± 0.012	0.191 ± 0.016	0.186 ± 0.009
B18	0.195 ± 0.010	0.212 ± 0.012	0.198 ± 0.010

**Table 6:** PMV test results of phase-change thermoregulatory fiber garments.

Sample Garment Number	Upper Garment PMV	Lower Garment PMV	Set PMV
B01	−3.28 ± 0.093	−2.51 ± 0.168	−2.86 ± 0.104
B02	−2.71 ± 0.085	−2.18 ± 0.131	−2.44 ± 0.086
B03	−2.21 ± 0.092	−1.83 ± 0.088	−1.83 ± 0.049
B04	−2.84 ± 0.104	−2.39 ± 0.048	−2.67 ± 0.120
B05	−2.36 ± 0.208	−1.91 ± 0.115	−1.97 ± 0.078
B06	−1.94 ± 0.198	−1.75 ± 0.069	−1.75 ± 0.088
B07	−3.40 ± 0.139	−2.64 ± 0.110	−2.9 ± 0.163
B08	−2.78 ± 0.110	−2.21 ± 0.201	−2.53 ± 0.071
B09	−2.53 ± 0.096	−2.12 ± 0.194	−2.32 ± 0.089
B10	−2.77 ± 0.101	−2.25 ± 0.249	−2.59 ± 0.127
B11	−2.65 ± 0.145	−2.02 ± 0.197	−2.38 ± 0.139
B12	−2.43 ± 0.092	−1.94 ± 0.208	−2.20 ± 0.155
B13	−3.47 ± 0.232	−2.85 ± 0.203	−3.28 ± 0.198
B14	−3.39 ± 0.122	−2.69 ± 0.100	−3.02 ± 0.074
B15	−3.28 ± 0.163	−2.46 ± 0.139	−2.81 ± 0.114
B16	−3.42 ± 0.237	−2.77 ± 0.094	−3.11 ± 0.081
B17	−3.42 ± 0.302	−2.58 ± 0.112	−2.93 ± 0.068
B18	−2.95 ± 0.298	−2.41 ± 0.181	−2.73 ± 0.098

## Data Availability

The raw data supporting the conclusions of this article/Supplementary Materials will be made available by the authors on request.

## Supplementary Materials

The following supporting information can be downloaded at: https://www.mdpi.com/article/10.3390/ma18235317/s1, Table S1: ANOVA for Between-Subjects Factors on Thermal Resistance. Table S2: Test of Between-Subjects Effects for Fabric PMV. Table S3: Results of Duncan’s Test for Fabric Thermal Resistance. Table S4: Results of Duncan’s Test for PMV. Table S5: Results of Duncan’s Test for PMV. Table S6: Results of Duncan’s Test for Thermal Resistance. Table S7: Fabric Thickness and Areal Density. Table S8: Normality and Homogeneity of Variances Tests.
