# Supplementary material for: Thermoregulatory Performance and Thermal Comfort Analysis of Phase-Change Fiber Seamless Knitted Fabrics
Source: Materials (Basel). 2025 Nov 25;18(23):5317. doi: 10.3390/ma18235317 (PMC12693357; doi:10.3390/ma18235317)
Supplement: Supplementary file 1 [file materials-18-05317-s001.zip › materials-3921030-supplementary.pdf]

materials

MDPI

Supporting Information

Thermoregulatory Performance and Thermal Comfort Analysis of Phase-Change Fiber Seamless Knitted Fabrics

Jingfeng Cheng [1], Lu Chang [2], Jiahui Fei [1], Zimin Jin [1,*] and Mingtao Zhao [3]

[1] College of Textile Science and Engineering (International Silk College), Zhejiang Sci-Tech University, Hangzhou, China; cjf0823@163.com (J.C.); feijiahui1999@163.com (J.F.)
[2] Shanghai Yijin Testing Technology Co., Ltd.; cherry@test-china.com
[3] Zhejiang Bangjie Holding Group Co., Ltd., Yiwu, China; mtzhao@bangjie.cn
* Correspondence: jinzimin@zstu.edu.cn

 https://doi.org/10.3390/ma18235317

Table S1. ANOVA for Between-Subjects Factors on Thermal Resistance.

Dependent Variable: Fabric Thermal Resistance Value							
Source	Dependent Variable	Type III Sum of Squares	df	Mean Square	F	Sig.	η^2p
Corrected Model	Clothing Ensemble	0.044[a]	17	0.003	12.805	< 0.001	0.858
	Upper Garment	0.043[b]	17	0.003	11.633	< 0.001	0.846
	Lower Garment	0.059[c]	17	0.003	10.690	< 0.001	0.835
Intercept	Clothing Ensemble	2.342	1	2.342	11554.279	< 0.001	0.997
	Upper Garment	2.258	1	2.258	10409.548	< 0.001	0.997
	Lower Garment	2.581	1	2.581	7884.025	< 0.001	0.995
A（Yarn Type）	Clothing Ensemble	0.029	5	0.006	28.874	< 0.001	0.800
	Upper Garment	0.026	5	0.005	23.768	< 0.001	0.768
	Lower Garment	0.034	5	0.007	20.668	< 0.001	0.742
B（Fabric Structure）	Clothing Ensemble	0.013	2	0.006	31.059	< 0.001	0.633
	Upper Garment	0.015	2	0.008	35.041	< 0.001	0.661
	Lower Garment	0.022	2	0.011	34.166	< 0.001	0.655
A（Yarn Type） * B（Fabric Structure）	Clothing Ensemble	0.002	10	0.000	1.121	0.374	0.237
	Upper Garment	0.002	10	0.000	0.883	0.557	0.197
	Lower Garment	0.003	10	0.000	1.015	0.450	0.220
Error	Clothing Ensemble	0.007	36	0.000			
	Upper Garment	0.008	36	0.000			
	Lower Garment	0.012	36	0.000			
Total	Clothing Ensemble	2.393	54				
	Upper Garment	2.309	54				
	Lower Garment	2.652	54				
Corrected Total	Clothing Ensemble	0.051	53				
	Upper Garment	0.051	53				
	Lower Garment	0.071	53				

a. $R^2 = 0.858$（Adjusted $R^2 = 0.791$）
b. $R^2 = 0.846$（Adjusted $R^2 = 0. 773$）
c. $R^2 = 0.835$（Adjusted $R^2 = 0.757$）

According to Cohen's guidelines, $\eta^2p \geq 0.01$ is considered a small effect, ≥ 0.06 a medium effect, and ≥ 0.14 a large effect.

Table S2. Test of Between-Subjects Effects for Fabric PMV.

Dependent variable: Fabric PMV value							
Source	Dependent Variable	Type III Sum of Squares	df	Mean Square	F	Sig.	η^2p
Corrected Model	Clothing Ensemble	9.745[a]	17	0.573	20.549	< 0.001	0.907
	Upper Garment	11.460[b]	17	0.674	14.288	< 0.001	0.871
Corrected Model	Lower Garment	5.711[c]	17	0.336	10.368	< 0.001	0.830
Intercept	Clothing Ensemble	357.590	1	357.590	12819.413	< 0.001	0.997
	Upper Garment	447.725	1	447.725	9489.046	< 0.001	0.996
	Lower Garment	287.318	1	287.318	8866.332	< 0.001	0.986
A（Yarn Type）	Clothing Ensemble	5.455	5	1.091	39.112	< 0.001	0.845
	Upper Garment	6.645	5	1.329	28.167	< 0.001	0.796
	Lower Garment	3.288	5	0.658	20.290	< 0.001	0.738
B（Fabric Structure）	Clothing Ensemble	3.575	2	1.787	64.079	< 0.001	0.781
	Upper Garment	3.687	2	1.844	39.073	< 0.001	0.685
	Lower Garment	2.164	2	1.082	33.388	< 0.001	0.650
A（Yarn Type） * B（Fabric Structure）	Clothing Ensemble	0.715	10	0.071	2.562	0.019	0.416
	Upper Garment	1.128	10	0.113	2.391	0.027	0.399
	Lower Garment	0.260	10	0.026	0.802	0.628	0.182
Error	Clothing Ensemble	1.004	36	0.028	0.028		
	Upper Garment	1.699	36	0.047	0.047		
Error	Lower Garment	1.167	36	0.032			

Total	Clothing Ensemble	368.339	54
	Upper Garment	460.884	54
	Lower Garment	294.196	54
Corrected Total	Clothing Ensemble	10.749	53
	Upper Garment	13.159	53
	Lower Garment	6.878	53

a. $R^2 = 0.907$（Adjusted $R^2 = 0.862$）
b. $R^2 = 0.870$（Adjusted $R^2 = 0.810$）
c. $R^2 = 0.830$（Adjusted $R^2 = 0.750$）

Table S3. Results of Duncan's Test for Fabric Thermal Resistance.

Multiple Comparisons for the Levels of YarnType.

1. Thermal Resistance Value of the Garment (Duncan[a,b])

Yarn Type	Number of Cases	Subset 1	Subset 2	Subset 3	Subset 4
cotton50 s	9	0.17356			
cotton32 s	9	0.18389			
3070Tempsolution32 s	9		0.20667		
5050Tempsolution32 s	9		0.21756	0.21756	
3070Tempsolution50 s	9			0.22889	0.22889
5050Tempsolution50 s	9				0.23889
Significance		0.132	0.113	0.100	0.145

2. Thermal Resistance of the Upper Garment (Duncan[a,b])

Yarn Type	Number of Cases	Subset 1	Subset 2	Subset 3
cotton50 s	9	0.17233		
cotton32 s	9	0.18000		
3070Tempsolution32 s	9		0.20667	
5050Tempsolution32 s	9		0.21467	
3070Tempsolution50 s	9		0.21833	
5050Tempsolution50 s	9			0.23500
Significance		0.277	0.121	1.000

3. Thermal Resistance Value of the Lower Garment (Duncan[a,b])

Yarn Type	Number of Cases	Subset 1	Subset 2	Subset 3
cotton50 s	9	0.18233		
cotton32 s	9	0.19167		
3070Tempsolution32 s	9		0.21933	
5050Tempsolution32 s	9		0.23000	
3070Tempsolution50 s	9		0.23233	
5050Tempsolution50 s	9			0.25600
Significance		0.281	0.158	1.000

Multiple Comparisons for the Levels of Fabric Structure.

4. Thermal Resistance Value of the Garment (Duncan[a,b])

Thermal Resistance Value of the Garment (Duncan[a,b])				
Fabric Structure	Number of Cases	Subset		
		1	2	3
Weft plain	18	0.18928		
1 + 1rib structure	18		0.20878	
1 + 3 mock rib structure	18			0.22667
Significance		1.000	1.000	1.000

5. Thermal Resistance of the Upper Garment (Duncan[a,b])

Thermal Resistance of the Upper Garment (Duncan[a,b])				
Fabric Structure	Number of Cases	Subset		
		1	2	3
Weft plain	18	0.18317		
1 + 1rib structure	18		0.20617	
1 + 3 mock rib structure	18			0.22417
Significance		1.000	1.000	1.000

6. Thermal Resistance Value of the Lower Garment (Duncan[a,b])

Thermal Resistance Value of the Lower Garment (Duncan[a,b])				
Fabric Structure	Number of Cases	Subset		
		1	2	3
Weft plain	18	0.19267		
1 + 1rib structure	18		0.22083	
1 + 3 mock rib structure	18			0.24233
Significance		1.000	1.000	1.000

Table S4. Results of Duncan's Test for PMV.

Multiple Comparisons for the Levels of YarnType

1. PMV of the Garment (Duncan[a,b])

PMV of the Garment (Duncan[a,b])					
Yarn Type	Number of Cases	Subset			
		1	2	3	4
cotton50s	9	−3.03667			
cotton32s	9	−2.92333			
3070Tempsolution32 s	9		−2.58333		
5050Tempsolution32 s	9			−2.39000	
3070Tempsolution50 s	9			−237667	
5050Tempsolution50 s	9				−2.13000
Significance		0.159	1.000	0.866	1.000

2. PMV of the Upper Garment (Duncan[a,b])

PMV of the Upper Garment (Duncan[a,b])					
Yarn Type	Number of Cases	Subset			
		1	2	3	4
cotton50 s	9	−3.3800			
cotton32 s	9	−3.2633			

3070Tempsolution32 s	9		−2.9033		
5050Tempsolution32 s	9		−2.7333	−2.7333	
3070Tempsolution50 s	9			−2.6167	
5050Tempsolution50 s	9				−2.3800
Significance		0.262	0.106	0.262	1.000

3. PMV of the Lower Garment (Duncan[a,b])

PMV of the Lower Garment (Duncan[a,b])					
Yarn Type	Number of Cases	Subset			
		1	2	3	4
cotton50 s	9	−2.6667			
cotton32 s	9	−2.5866			
3070Tempsolution32 s	9		−2.32333		
5050Tempsolution32 s	9		−2.17667	−2.17667	
3070Tempsolution50 s	9			−2.07000	
5050Tempsolution50 s	9			−2.01667	
Significance		0.352	0.092	0.082	

Multiple Comparisons for the Levels of Fabric Structure.

4. PMV of the Garment (Duncan[a,b])

PMV of the Garment (Duncan[a,b])				
Fabric Structure	Number of Cases	Subset		
		1	2	3
Weft plain	18	−2.90167		
1 + 1rib structure	18		−2.54500	
1 + 3 mock rib structure	18			−2.27333
Significance		1.000	1.000	1.000

5. PMV of the Upper Garment (Duncan[a,b])

PMV of the Upper Garment (Duncan[a,b])				
Fabric Structure	Number of Cases	Subset		
		1	2	3
Weft plain	18	−3.19667		
1 + 1rib structure	18		−2.88500	
1 + 3 mock rib structure	18			−2.55667
Significance		1.000	1.000	1.000

6. PMV of the Lower Garment (Duncan[a,b])

PMV of the Lower Garment (Duncan[a,b])				
Fabric Structure	Number of Cases	Subset		
		1	2	3
Weft plain	18	−2.5700		
1 + 1rib structure	18		−2.2650	
1 + 3 mock rib structure	18			−2.0850
Significance		1.000	1.000	1.000

The results of Duncan's test ($p < 0.05$) are presented in Table S4 using letter labels. Groups sharing a common letter are not statistically significantly different.

Table S5. Results of Duncan's Test for PMV.

Factor	Level	Upper Garment PMV	Lower Garment PMV	Garment PMV
Yarn Type	5050Tempsolution yarn 50 s	a	a	a
	5050Tempsolution yarn 32 s	b	a	b
	3070Tempsolution yarn 50 s	bc	ab	b
	3070Tempsolution yarn 32 s	c	b	c
	Cotton Yarn 50 s	d	c	d
	Cotton Yarn 32 s	d	c	d
Fabric Structure	Weft Plain	c	c	c
	1 + 1 rib structure	b	b	b
	1 + 3 mock rib structure	a	a	a

Table S6. Results of Duncan's Test for Thermal Resistance.

Factor	Level	Upper Garment	Lower Garment	Garment
Yarn Type	5050Tempsolution yarn 50 s	a	a	a
	5050Tempsolution yarn 32 s	b	b	bc
	3070Tempsolution yarn 50 s	b	b	ab
	3070Tempsolution yarn 32 s	b	b	c
	Cotton Yarn 50 s	c	c	d
	Cotton Yarn 32 s	c	c	d
Fabric Structure	Weft Plain	c	c	c
	1 + 1 rib structure	b	b	b
	1 + 3 mock rib structure	a	a	a

Table S7. Fabric Thickness and Areal Density.

Sample Number	Grammage/($g{\cdot}m^{-2}$)	Thickness/mm
B01	240.35	0.73
B02	304.6	1.08
B03	398.84	1.75
B04	247.81	0.73
B05	312.97	1.04
B06	401.19	1.72
B07	269.04	0.78
B08	337.64	1.09
B09	442.03	1.86
B010	278.40	0.75

B011	349.72	1.09
B012	456.67	1.85
B013	229.13	0.74
B014	296.53	1.10
B015	376.95	1.72
B016	252.17	0.78
B017	321.32	1.15
B018	451.77	2.00

Table S8. Normality and Homogeneity of Variances Tests.

**Variable**	**Test**	**Statistic**	**p-Value**
Thermal Resistance	Shapiro-Wilk		
	Clothing Ensemble	W = 0.970	P = 0.194
	Upper Garment	W = 0.960	P = 0.066
	Lower Garment	W = 0.966	P = 0.123
	Levene's Test		
	Clothing Ensemble	F = 0.911	P = 0.568
	Upper Garment	F = 0.605	P = 0.865
	Lower Garment	F = 0.944	P = 0.535
PMV	Shapiro-Wilk		
	Clothing Ensemble	W =0.974	P = 0.298
	Upper Garment	W =0.972	P = 0.232
	Lower Garment	W =0.971	P = 0.221
	Levene's Test		
	Clothing Ensemble	F = 1.285	P = 0.286
	Upper Garment	F = 0.958	P = 0.520
	Lower Garment	F = 1.069	P = 0.417
